# Intelligent Algorithm-Based Magnetic Resonance Imaging in Radical Gastrectomy under Laparoscope

**DOI:** 10.1155/2021/1701447

**Published:** 2021-09-14

**Authors:** Wenkui Mo, Cansong Zhao

**Affiliations:** ^1^Department of Thoracic Surgery, Cancer Hospital of the University of Chinese Academy of Sciences (Zhejiang Cancer Hospital), Hangzhou 310022, Zhejiang, China; ^2^Department of General Surgery, Zhuji People's Hospital of Zhejiang, Zhuji 311800, Zhejiang, China

## Abstract

The study focused on the influence of intelligent algorithm-based magnetic resonance imaging (MRI) on short-term curative effects of laparoscopic radical gastrectomy for gastric cancer. A convolutional neural network- (CNN-) based algorithm was used to segment MRI images of patients with gastric cancer, and 158 subjects admitted at hospital were selected as research subjects and randomly divided into the 3D laparoscopy group and 2D laparoscopy group, with 79 cases in each group. The two groups were compared for operation time, intraoperative blood loss, number of dissected lymph nodes, exhaust time, time to get out of bed, postoperative hospital stay, and postoperative complications. The results showed that the CNN-based algorithm had high accuracy with clear contours. The similarity coefficient (DSC) was 0.89, the sensitivity was 0.93, and the average time to process an image was 1.1 min. The 3D laparoscopic group had shorter operation time (86.3 ± 21.0 min vs. 98 ± 23.3 min) and less intraoperative blood loss (200 ± 27.6 mL vs. 209 ± 29.8 mL) than the 2D laparoscopic group, and the difference was statistically significant (*P* < 0.05). The number of dissected lymph nodes was 38.4 ± 8.5 in the 3D group and 36.1 ± 6.0 in the 2D group, showing no statistically significant difference (*P* > 0.05). At the same time, no statistically significant difference was noted in postoperative exhaust time, time to get out of bed, postoperative hospital stay, and the incidence of complications (*P* > 0.05). It was concluded that the algorithm in this study can accurately segment the target area, providing a basis for the preoperative examination of gastric cancer, and that 3D laparoscopic surgery can shorten the operation time and reduce intraoperative bleeding, while achieving similar short-term curative effects to 2D laparoscopy.

## 1. Introduction

Gastric carcinoma is a common malignant tumor of the digestive tract. According to the *Global Cancer Statistics 2018*, there are approximately 18.19 million new cancer cases worldwide and 9.6 million cancer deaths. The death rate of gastric cancer ranks third among all cancers, and patients suffering from gastric cancer account for 8.2% of the total cancer cases, second only to lung cancer (18.4%) and colorectal cancer (9.2%). Asia is a region with high incidence of gastric cancer, especially Mongolia, South Korea, Japan, and China. According to Chen et al. [1], there are 400,000 new cases of gastric cancer in China each year, accounting for 42% of the global new cases of gastric cancer. Gastric cancer usually originates from gastric antrum, gastric varices, and cardia. The prognosis of patients with gastric cancer varies depending on the clinical stage. Computed tomography (CT) and magnetic resonance imaging (MRI) examinations can evaluate tumor staging and the curative effects [2]. MRI images have high resolution for soft tissue and no ionizing radiation, and images from various angles can be obtained. In recent years, MRI rapid breath-holding sequence, respiratory gating technology, and the development of various gastrointestinal agents have significantly improved the image quality of the gastrointestinal tract [3]. MRI has demonstrated a high accuracy rate in the diagnosis and staging of gastric cancer, and it has been widely used in the diagnosis of gastric cancer. It can clearly show the location, range, shape, and size of gastric cancer [4]. Laparoscopy is a minimally invasive surgery in which doctors operate through a small incision in the abdomen, which reduces the damage to the patient and greatly reduces the chance of postoperative adhesions. Except for distal gastrectomy, laparoscopic gastrectomy is also applied to proximal gastrectomy and total gastrectomy [5]. With the development of laparoscopy and endoscopy technology, 3D high-definition laparoscope has emerged. It can provide three-dimensional vision enabling precise spatial positioning, overcoming the inability to determine the anatomical level of the currently widely used 2D laparoscope [6].

MRI images of advanced gastric cancer are characterized by irregular thickening of the stomach wall or soft tissue masses, with or without ulcers, and gastric cavity deformation and stenosis. In order to better identify the lesion, it is necessary to segment the image. The segmentation of gastric cancer images is generally performed by experts manually. This depends on the expert's medical knowledge and experience and has strong subjectivity. As a result, different experts may have different conclusions [7]. Additionally, manual segmentation is time-consuming, labor-intensive, and unable to deal with large amount medical information. Therefore, the use of image segmentation algorithms for automatic or semiautomatic segmentation of tumors can greatly reduce the workload of doctors. Most image processing algorithms extract features based on specific formulas, which require a manual design process, and it will be affected by the subjective perceptions of the designer. Convolutional neural networks (CNN) are now widely used in the field of medical image processing [8]. CNNs do not require human involvement, and automatically learn features for specific tasks through neural networks. Automatic tumor segmentation based on CNN is an important research direction in the field of medical image processing [9].

There is much research applying CNN to segment brain tumors, but there are few studies on the segmentation of MRI images of gastric tumors. In this regard, in this study, a CNN-based algorithm was used for image segmentation, to compare the clinical effects of 3D and 2D laparoscopic radical gastrectomy.

## 2. Materials and Methods

### 2.1. Research Subjects

In this study, 158 patients undergoing radical gastrectomy admitted to the hospital from June 2018 to September 2020 were selected, including 87 males and 71 females, aged 32–77 years old. They were randomly divided into 2D laparoscopy group and 3D laparoscopy, with 79 cases in each group. The differences in general data such as gender and age between the two groups of patients were not statistically significant (*P* > 0.05), and they were comparable. This study has been approved by the ethics committee of the hospital. All patients and their families were aware of the study and signed the informed consent form.

 
*Inclusion Criteria.* (i) Patients who were pathologically diagnosed as primary gastric cancer under gastroscopy; (ii) patients who can tolerate continuous gastric filling and can eat independently; (iii) patients with good condition who can have MRI examination; (iv) patients without MRI contraindications, such as cardiac pacemakers, neurostimulators, aneurysm clips, insulin pumps, and cochlear implants; (v) patients who fully understood this research and signed the informed consent form; (vi) patients with complete MRI data.
 
*Exclusion Criteria.* (i) Patients with other neoplastic diseases at the same time, such as gastric metastatic tumors; (ii) patients accompanied by pyloric obstruction or other types of gastrointestinal obstruction; (iii) patients with mental illness; (iv) those who were intolerant of contrast agents; (v) patients with severe heart and lung diseases; (vi) patients with liver and kidney dysfunction.

### 2.2. MRI Examination

Before the MRI examination, the patient should fast for 8–10 hours. 20 minutes before the examination, 10–20 mg of anisodamine was injected intramuscularly to reduce gastrointestinal motility, and then the patient should drink 800–1000 mL of warm water to fill the stomach. The equipment used was GE Hi Speed 1.5T MR scanner. The scanning sequence was diffusion-weighted imaging, and the scanning positions included transverse plain scanning, coronal scanning, and sagittal scanning.

### 2.3. CNN-Based Image Segmentation

**Figure 1:**
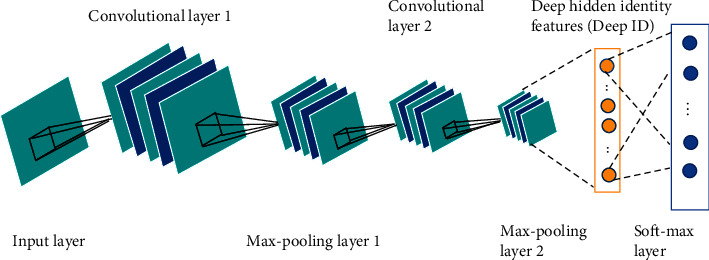
Structure diagram of the CNN.

CNNs can automatically learn abstract features from images for classification. Its network structure is shown in Figure 1. Usually, it comprises the input layer, the convolutional layer, the subsampling layer, the fully connected layer, and the output layer.

CNN independently estimates the voxel label according to the neighborhood and context information of each voxel in the image and realizes feature extraction by cascading a series of convolution operations. 3D CNN uses a convolution kernel to perform convolution operations on the original three-dimensional data, the input layer, and the convolution kernel. After adding the bias term, the output feature map is obtained through a nonlinear activation function calculated as follows:

(1) $$\begin{array}{c} y_{l-1}^{a}=f\left(\sum k_{l}^{a,b}*y_{l-1}^{b}+m_{l}^{a}\right). \end{array}$$

Each convolution kernel *k*_*l*_^*a*,*b*^ is the hidden weight, *a* refers to the number of convolution kernels, that is, the output feature *dimension*, *b* is the input feature dimension, *y*_*l*−1_^*b*^ represents the output three-dimensional feature map, *m*_*l*_^*a*^ is the offset term, ^*∗*^ represents the *convolution* operation, and *f* is a nonlinear activation function. The activation function is to map nonlinear changes of the features in the neural network to a specific space. Common activation functions include the following:

(1) Sigmoid, defined as
  (2) $$\begin{array}{c} \text{sigmoid}\left(x\right)=\frac{1}{1+e^{-x}}. \end{array}$$
(2) Linear rectified unit (ReLu), defined as
  (3) $$\begin{array}{c} f\left(x\right)=\max\left(0,x\right). \end{array}$$
(3) Leaky rectified linear function (LReLU), defined as
  (4) $$\begin{array}{c} f\left(x\right)=\left\{\begin{array}{cc} x, & x\geq 0,\\ m_{i}x, & x\leq 0. \end{array}\right. \end{array}$$
(4) Hyperbolic tangent function, defined as

(5) $$\begin{array}{c} \tanh\left(x\right)=\frac{\sin\left(x\right)}{\cosh\left(x\right)}=\frac{e^{x}-e^{-x}}{e^{x}+e^{-x}}. \end{array}$$

The classification layer uses a normalized exponential function (Softmax), which is defined as follows.

(6) $$\begin{array}{c} S_{i}=\frac{e^{V_{i}}}{\sum_{i}^{C}e^{V_{i}}}, \end{array}$$

where *V*_*i*_ refers to the output of the previous output unit, *i* refers to the index number of the predicted category, and *C* is the number of categories. *S*_*i*_ represents the ratio of the index of the current predicted category to the sum of the element indices of all categories, and the output value can be converted into the predicted probability of the corresponding category through Softmax.

### 2.4. Surgical Methods

The two groups had the same preoperative preparations, and both groups fasted for 12 hours before the operation. Tracheal intubation was performed for general anesthesia, and the patient was in a supine position. Intra-abdominal pressure was maintained at 1.6∼2.0 kPa. The two groups had radical gastrectomy under 3D and 2D laparoscopic systems, respectively.

### 2.5. Evaluation of the Image Segmentation Effects

The performance of the algorithm is quantitatively evaluated factoring into the Dice similarity coefficient (DSC) and sensitivity. DSC describes the degree of overlap between the segmentation result and the true value, and the sensitivity represents the proportion of the correct segmentation result to the true value. They are defined as follows.

(7) $$\begin{array}{c} \text{DSC}=\frac{2\left|A\cap B\right|}{\left|A\right|+\left|B\right|}\times 100\%,\\ \text{sensitivity}=\frac{\left|A\cap B\right|}{\left|A\right|}, \end{array}$$

where *A* is the manual segmentation result by the expert and *B* is the segmentation result of the algorithm. *A*∩*B* is the intersection of *A* and *B*. A DSC index closer to 1 means that the two images are more similar and possess better segmentation effects. Then, the algorithm proposed by Havaei et al. [10] and Pereira et al. [11] were introduced to compare with the algorithm in this study.

### 2.6. Surgery Evaluation Index

The two groups were compared for operation time, intraoperative blood loss, number of dissected lymph nodes, postoperative exhaust time, time to get out of bed, postoperative hospital stay, and postoperative complications.

### 2.7. Statistical Methods

SPSS 20.0 was used to process the data. Measurement data were expressed by mean ± standard deviation ($\bar{x}$ ± *s*), and *t*-test was used. The *count* data adopted the chi-square test. *P* < 0.05 was the threshold for significance.

## 3. Results

### 3.1. Image Processing Results

**Figure 2:**
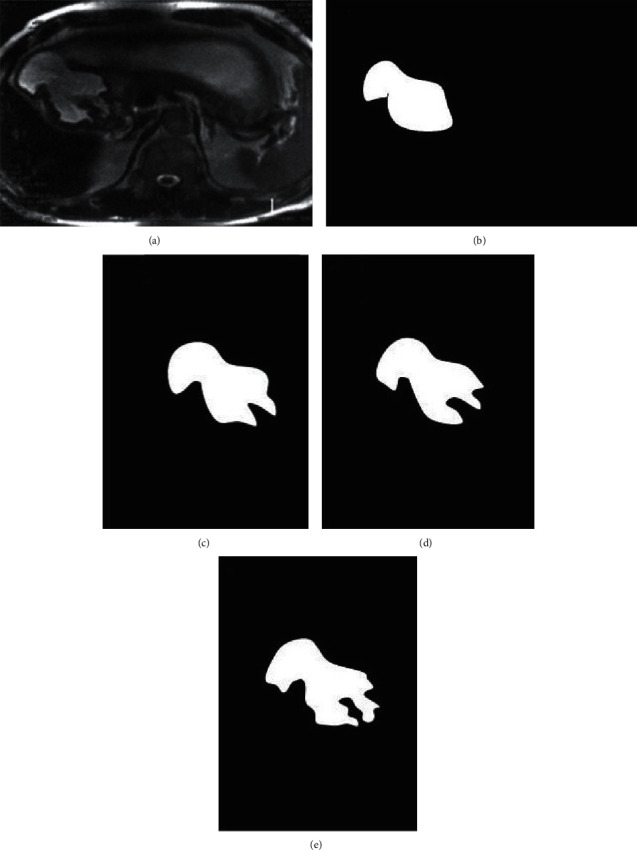
Comparison of segmentation results: (a) original image; (b) the result of manual segmentation by experts; (c) the segmentation result of convolutional neural network; (d) the segmentation result of Havaei algorithm; (e) the segmentation result of Pereira's algorithm.

Figure 2 showed the segmentation results by different methods.  Figure 2(a) was the original image, Figure 2(b) was the result of manual segmentation by experts, Figure 2(c) was the segmentation result by CNN, and Figures 2(d) and 2(e) were the results by the algorithms proposed by Havaei and Pereira, respectively. It was noted that the segmentation result of the CNN in this study was better, the outline was clear, and the accuracy was higher. The segmentation result of Figure 2(d) was relatively poor.

### 3.2. Evaluation of Image Segmentation Results

**Figure 3:**
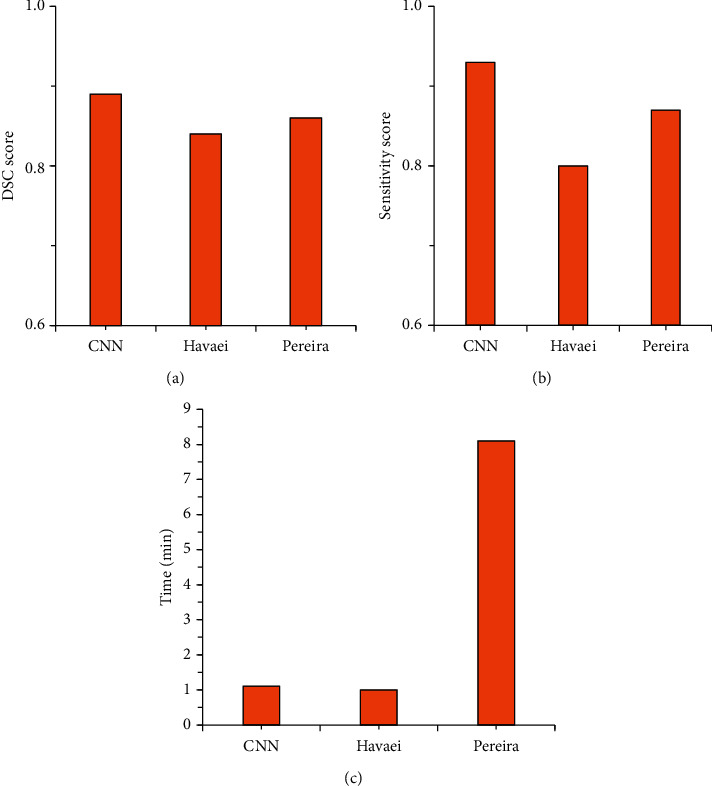
Comparison of DSC, sensitivity, and running time of the three algorithms. (a) DSC coefficient; (b) sensitivity; (c) running time.

In order to quantitatively compare the segmentation performance of the CNN in this study, the three algorithms were compared for the DSC, sensitivity, and the average running time.  Figure 3(a) is the similarity coefficient of the three algorithms, Figure 3(b) is the sensitivity, and Figure 3(c) is the time taken to process an image. The average time of the algorithm in this study to segment an image was approximately 1.1 minutes, the DSC was 0.89, and the sensitivity was 0.93. Compared with Havaei's algorithm, the sensitivity was increased by 13%, and the time was basically the same. Compared with Pereira's algorithm, the segmentation speed, DSC, and sensitivity of the CNN in the study were significantly improved.

### 3.3. Comparison of Surgical Indexes of 3D and 2D Laparoscopic Radical Gastrectomy

**Figure 4:**
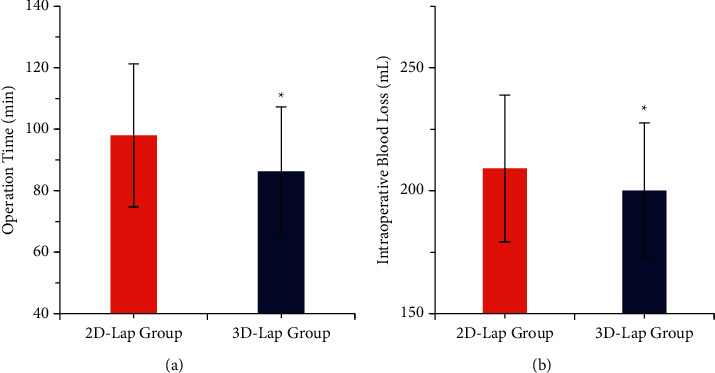
Comparison of operation time and intraoperative blood loss between the two groups. (a) Operation time. (b) Intraoperative blood loss; ^*∗*^*P* < 0.05. The difference between groups was statistically significant.

**Figure 5:**
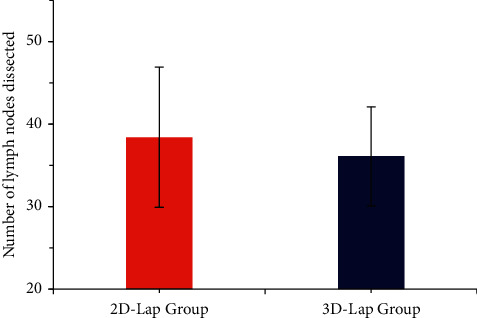
Comparison of the number of dissected lymph nodes between the two groups.

The two groups were compared for the operation time, blood loss, and the number of dissected lymph nodes, and the results were shown in Figures 4 and 5. The operation time (98 ± 23.3 min) and intraoperative blood loss (209 ± 29.8 mL) of the 3D laparoscopic group were less than those of the 2D laparoscopic group (86.3 ± 21.0 min, 200 ± 27.6 mL), and the difference was statistically different (*P* < 0.05), the number of dissected lymph nodes in the 3D laparoscopic group was 38.4 ± 8.5, and that in the 2D group was 36.1 ± 6.0, showing no statistical difference (*P* > 0.05).

### 3.4. Comparison of Recovery Indexes after 3D and 2D Laparoscopic Radical Gastrectomy

**Figure 6:**
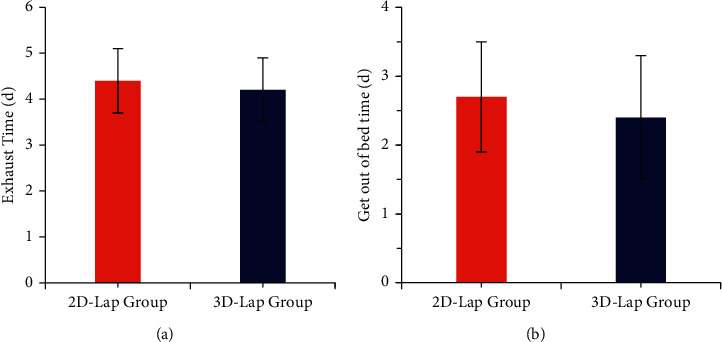
Comparison of the postoperative exhaust time and the time of getting out of bed between the two groups of patients. (a) Postoperative exhaust time. (b) The time of getting out of bed.

**Figure 7:**
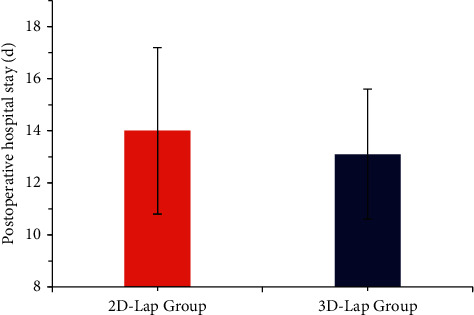
Comparison of postoperative hospital stay between the two groups.

The two groups were compared for postoperative exhaust time, the time of getting out of bed, and the postoperative hospital stay. The postoperative exhaust time of the 3D laparoscopic group was 4.2 ± 0.7d, the time to get out of bed was 2.4 ± 0.9d, and the postoperative hospital stay was 13.1 ± 2.5d. In the 2D laparoscopic group, the exhaust time was 4.4 ± 0.7d, the time to get out of bed was 2.7 ± 0.8d, and the postoperative hospital stay was 14 ± 3.2d. The exhaust time, time to get out of bed, and postoperative hospital stay in the 3D laparoscopic group were lower than those in the 2D laparoscopic group, but the difference was not statistically significant (*P* > 0.05) (Figures 6 and 7).

### 3.5. Comparison of Complications after 3D and 2D Laparoscopic Radical Gastrectomy

The types and incidence of postoperative complications of the two groups are shown in Table 1. The incidence of postoperative complication rate in the 2D laparoscopic group was 7.6%, and that of the 3D laparoscopic group was 5.1%, showing no statistically significant differences (*P* > 0.05).

## 4. Discussion

Gastric cancer is a malignant tumor with high morbidity and mortality. With the improvement of living standards and the increase of life pressure, the incidence of gastric cancer is increasing year by year. Surgical resection is the key to comprehensive treatment of gastric cancer. Traditional open surgery has the advantages of clear vision and convenient operations, but it leads to large trauma, large intraoperative bleeding, and slow incision healing [12]. In recent years, the effectiveness and safety of laparoscopic or laparoscopic-assisted radical gastrectomy in advanced gastric cancer are gradually being recognized. In other words, laparoscopic radical gastrectomy has become a main surgical method for the treatment of gastric cancer in the world [13]. However, traditional laparoscopy also has some limitations. Traditional laparoscopy provides a two-dimensional field of vision and lacks depth and direction recognition. Consequently, it is inconvenient to position lesions and acquire spatial depth information, and sometimes, it is difficult to identify the front and back relationship of surgical instruments [14, 15]. 3D laparoscopy builds a three-dimensional space, providing larger magnification, which improves the accuracy and efficiency of the operation and reduced additional discomfort such as visual fatigue [16, 17]. Studies have shown that, compared with traditional laparoscopic surgery, three-dimensional laparoscopic surgery can shorten the operation time, reduce intraoperative blood loss, and shorten the learning curve for young doctors [18, 19].

The histopathological classification and degree of differentiation of gastric cancer are important characteristics in the selection of treatment plans and prognostic evaluation of gastric cancer. MRI examination is a noninvasive examination method without radiation damage. It can accurately display morphological features of gastric cancer through multidirectional imaging, and it has good contrast for soft tissue and thus can clearly show gastric cancer lesions [20]. Although there are a variety of image segmentation algorithms, it is difficult to get good results because the information in medical images is complex, the grayscale distribution is uneven, the noise is large, and the organs and tissues are prone to deformation [21]. The CNN can automatically learn abstract features from the image for later analysis [22, 23].

In this study, a CNN-based algorithm was used to segment MRI images of gastric cancer. The segmentation results were compared with the expert manual segmentation results, and it was found to have high accuracy, and the outline was clear. The algorithms proposed by Havaei and Pereira were introduced for comparison. It was found that the algorithm of the study had higher sensitivity, DSC, and faster segmentation speed versus these two algorithms. The automatic segmentation algorithm in this study can reduce the burden on doctors and provide a basis for the diagnosis of gastric cancer staging. Also, the 2D laparoscopy and 3D laparoscopy were compared for the short-term curative effects on radical gastrectomy. The results showed that compared with 2D laparoscopy, 3D laparoscopy has shorter surgical time and less blood loss. The stereoscopic vision provided by the 3D laparoscope exposes blood vessels, reducing the difficulty dissecting lymph nodes in complex parts, such as the splenic hilum, thereby improving the efficiency of the operation, reducing the traction damage to the blood vessels, and reducing the blood loss. In terms of postoperative exhaust time, time to get out of bed, postoperative hospital stay, and postoperative complications, there was no statistically significant difference between 3D and 2D laparoscopic surgery patients, indicating that 3D laparoscopic surgery was comparable to traditional 2D laparoscopic surgery in safety.

## 5. Conclusion

In this study, a CNN-based algorithm was used to segment the MRI images of gastric cancer. It was found to have high accuracy and can speed up the processing speed of clinical images, providing a basis for the diagnosis and treatment of gastric cancer. Compared with traditional 2D laparoscopic radical gastrectomy, 3D laparoscopic surgery can shorten the operation time and reduce the amount of intraoperative blood loss, while achieving similar short-term clinical effects. However, this study still has some limitations. The sample size is small, which will reduce the power of the study, and the patients are not divided into more detailed groups according to the different surgical procedures such as total gastrectomy, distal gastrectomy, and proximal gastrectomy. In the follow-up, an expanded sample size is needed to strengthen the findings of the study.

## Figures and Tables

**Table 1:** Comparison of postoperative complications between the two groups.

	Postoperative complications		
	Pancreatic fistula	Anastomotic leakage	Postoperative bowel obstruction
2D-lap group	3	2	1
3D-lap group	2	1	1

## Data Availability

The data used to support the findings of this study are available from the corresponding author upon request.
